# EXAMINING PUBLIC AWARENESS OF AGEIST TERMS ON TWITTER: CONTENT ANALYSIS

**DOI:** 10.1093/geroni/igae098.1017

**Published:** 2024-12-31

**Authors:** Kristine Mulhorn

**Affiliations:** Drexel University, Philadelphia, Pennsylvania, United States

## Abstract

Following the efforts of the World Health Organization, the Centers for Disease Control and Prevention, and the Gerontological Society of America to raise awareness about ageist language and propose appropriate terms to denote the older adult population, there was a recognized hostile discourse on social media towards those most vulnerable to the COVID-19 pandemic. This study aimed to understand the prevalence and situated use of ageist terms on Twitter. We collected 60.32 million tweets between March and July 2020 containing terms related to COVID-19. We then conducted a mixed methods study comprising a content analysis and a descriptive quantitative analysis. There were a total of 58,930 tweets contained the ageist terms “old people” or “elderly.” The more appropriate term “older adult” was found in 11,328 tweets. Twitter users used ageist terms (eg, “old people” and “elderly”) to criticize ageist messages (17/60, 28%), showing a lack of understanding of appropriate terms to describe older adults. Highly hostile ageist content against older adults came from tweets that contained the derogatory terms “old people” (22/30, 73%) or “elderly” (13/30, 43%). The public discourse observed on Twitter shows a continued lack of understanding of appropriate terms to use when referring to older adults. Effort is needed to eliminate the perpetuation of ageist messages that challenge healthy aging. Our study highlights the need to inform the public about appropriate language use and ageism.

